# Mouse arylamine *N*-acetyltransferase 2 (*Nat2*) expression during embryogenesis: a potential marker for the developing neuroendocrine system

**DOI:** 10.1080/13547500701673529

**Published:** 2008-01-10

**Authors:** Larissa Wakefield, Valerie Cornish, Hilary Long, Akane Kawamura, Xiaoyan Zhang, David W. Hein, Edith Sim

**Affiliations:** 1 Department of Pharmacology, University of Oxford, Mansfield Road, Oxford, UK; 2 Department of Pharmacology and Toxicology and James Graham Brown Center, University of Louisville School of Medecine, Kentucky, USA

**Keywords:** Arylamine N-acetyltransferase/NAT, adrenal, embryo, breast cancer

## Abstract

Arylamine *N*-acetyltransferase (*NAT*) genes in humans and in rodents encode polymorphic drug metabolizing enzymes. Human NAT1 (and the murine equivalent mouse Nat2) is found early in embryonic development and is likely to have an endogenous role. We report the detailed expression of the murine gene (*Nat2*) and encoded protein in mouse embryos, using a transgenic mouse model bearing a *lacZ* transgene inserted into the coding region of mouse *Nat2*. In mouse embryos, the transgene was expressed in sensory epithelia, epithelial placodes giving rise to visceral sensory neurons, the developing pituitary gland, sympathetic chain and urogenital ridge. In *Nat2*^+/+^ mice, the presence and activity of Nat2 protein was detected in these tissues and their adult counterparts. Altered expression of the human orthologue in breast tumours, in which there is endocrine signalling, suggests that human *NAT1* should be considered as a potential biomarker for neuroendocrine tissues and tumours.

## Introduction

The arylamine *N*-acetyltransferases (NATs) are clinically important in terms of their role in metabolism of medicinal and environmental arylamines (Sim et al. 2007). These genes are also implicated in cancer of the breast (Adam et al. 2003), bladder, colon and prostate (Hein 2002). In humans, and in rodent models, the *NAT* genes are polymorphic, and *in vitro* assays of acetylation by NAT enzymes using arylamine substrates have been used to define the acetylation status of individuals (Weber & Hein 1985). *NAT* genes comprise polymorphic multigene families located at syntenic positions on chromosome 8 in mouse and man (Boukouvala & Fakis 2005), with mouse used widely to model human *NAT* (Hein et al. 1997, Sugamori et al. 2003). Human NAT2 was initially identified as a determinant of isoniazid metabolism and is largely active in the liver and intestine, indicating a role primarily in xenobiotic metabolism, as is the mouse Nat1 enzyme (Sim et al. 2007). On the basis of C-terminus sequence identity, substrate specificity and expression profile (Kawamura et al. 2005), human *NAT1* is orthologous to mouse *Nat2*. Human *NAT1* and mouse *Nat2* are expressed in a wide range of adult tissues and active during embryogenesis (McQueen et al. 2003), reviewed in (Boukouvala & Fakis 2005). Although endogenous roles relating to folate metabolism (Minchin 1995), acetyl coenzyme A or lipid homeostasis (Richards et al. 2004) have been postulated, the exact nature of the endogenous role for human NAT1 is still not proven. To further understand the role of human NAT1 in endogenous metabolism, we used homologous recombination in a mouse model to achieve functional deletion of *Nat2* (orthologous to human *NAT1*) in mice, by insertion of *lacZ* into the mouse *Nat2*-coding region (Cornish et al. 2003). This generated a mouse *Nat2*-knockout model (*Nat2*^−/−^) lacking Nat2-directed *N*- and *O*-acetyltransferase activities (Cornish et al. 2003, Loehle et al. 2006). As with mice lacking both *Nat1* and *Nat2* genes (Sugamori et al. 2003, 2006) *Nat2*^−/−^ mice do not display an obvious phenotype, however, the *Nat2* null allele has a gender-specific effect, giving rise to a sexual bias in *Nat2* allelic inheritance. In the homozygous state, the *Nat2* null allele gives rise to a male bias in the A/J strain (Cornish et al. 2003).

Although widely described as ubiquitously expressed, mouse *Nat2* expression is non-uniform within a given tissue. Within the cerebellum, for example, *Nat2* is robustly expressed in the Purkinje cells (Stanley et al. 1998). The present study uses detailed histochemical analysis and enzymatic evidence to provide a novel link between sites of robust *Nat2* expression through embryogenesis and on into adulthood and identifies *Nat2* as a biomarker of neuroendocrine development.

## Methods

### Nat2 transgenic mouse maintenance and breeding

All work involving animals was carried out according to the UK Animals (Scientific Procedures) Act of 1986 under license from the UK Home Office.

The generation of a stable *Nat2* knockout line of mice by targeted insertion of a *lacZ*-containing cassette, has been described (Cornish et al. 2003). Essentially, a *TAG3/IRES/lacZ/loxP/neo/loxP* reporter ablation cassette was inserted into the Bgl II site in the mouse *Nat2* coding region, an MCl-thymidine kinase dimer negative selection cassette was appended, and the resulting construct used to generate a null allele of mouse *Nat2* by homologous recombination in 129/Ola ES cells. The *Nat2* null allele was bred onto a C57Bl/6 background by backcrossing *Nat2*^+/−^ males to C57Bl/6 females (supplied by Harlan UK) over eleven generations. *Nat2*^−/−^ and *Nat2*^+/+^ animals used for analysis were generated by mating *Nat2*^+/−^ male and *Nat2*^+/−^ females derived from backcross matings. On weaning, ear biopsies were taken for genotyping. DNA was isolated using Sigma GenElute mammalian genomic DNA miniprep kit. Primers used for genotyping of the *Nat2* null allele were Neo-T (forward) and mNat2-910 (reverse) with mNat2-1 (forward) and mNat2-910 (reverse) used to detect the wild-type *Nat2* allele essentially as described (Cornish et al. 2003). MgCl_2_ was used at a final concentration of 2 mM; polymerase chain reaction conditions were initial denaturation 5 min at 95°C, denaturation at 94°C 30 s, annealing at 56°C 30 s, elongation at 72°C 45 s, 35 cycles.

To obtain embryos, timed matings were established, with noon on the day of the vaginal plug designated as e 0.5. Pregnant dams were killed by cervical dislocation. Embryos and associated yolk sacs were dissected from the uterus into ice-cold 10 mM potassium phosphate pH 7.5, 145 mM NaCl (phosphate-buffered saline) containing 4% paraformaldehyde. DNA for genotyping embryos was isolated from yolk sacs as described above.

### Preparation of protein samples for immunoblotting and acetylation assays

Tissues were dissected from adult animals immediately following cervical dislocation, trimmed, washed briefly in phosphate-buffered saline and either snap frozen and stored in liquid nitrogen or used for preparation of homogenates. Tissues were homogenized in three volumes of buffer: 20 mM KCl, 10 mM potassium phosphate buffer pH 7.5, 1.0 mM EDTA, 1.0 mM DTT, 0.5 mM Pefabloc protease inhibitor (Pentapharm, Basel, Switzerland), using an Ultraturax T25 tissue homogenizer, or, in the case of embryonic mouse tissues, using glass Dounce homogenizers. Samples were prepared for Western blotting or acetylation activities, and protein concentrations determined as described previously (Smelt et al. 2000). For each genotype and each sex, tissues were dissected from 8-week-old mice. Tissue homogenates were assayed for their ability to acetylate *para*-aminobenzoic acid (pABA) essentially as described (Smelt et al. 2000) or were used for immunoblotting. An aliquot of liver homogenate made from pooled tissues taken from six adult male C57Bl/6 *Nat2*^+/+^ mice was included in each acetylation assay as an internal standard (Smelt et al. 2000).

For immunoblotting, filters were probed for 1 h at 20°C with rabbit polyclonal antiserum 184 (used at 1:4000), raised against the C-terminal dodecapeptide of human NAT1, (identical to that of mouse Nat2), and conjugated to bovine serum albumin, as described (Cornish et al. 2003). The amounts of protein loaded into each well was confirmed by stripping the nitrocellulose filters for 30 min at 50°C in 100 mM 2-mercaptoethanol, 2% (w/v) SDS, 62.5 mM Tris–HCl pH 6.7, followed by two 10-min washes in Tris-buffered saline (300 mM NaCl, 50 mM Tris pH 7.6, 0.1% Tween 20), and overnight incubation in 3% non-fat dried milk powder diluted in Tris-buffered saline to block, before reprobing with β-actin polyclonal antibodies (A5060 Sigma-Aldrich, UK), at a dilution of 1:500. Bound antibody was visualized using the ECL Plus luminescent detection system (Amersham plc, UK).

### Whole-mount staining for β-galactosidase activity

Embryos were isolated as above then transferred to formaldehyde/gluteraldehyde buffer (2% formaldehyde, 0.2% gluteraldehyde, 0.02% NonidetP-40, 0.01% sodium desoxycholate in phosphate-buffered saline) at room temperature for 10 min. After fixation, tissues were rinsed four times in phosphate-buffered saline, then stained in the dark overnight at 37°C in x-gal stain solution (5 mM K_3_Fe(CN)_6_, 5 mM K_4_Fe(CN)_6_, 1 mg ml^−1^ 5-bromo-4-chloro-3-indolyl-β-D-galactopyranoside (x-gal), 2 mM MgCl_2_, 0.02% NP-40, 0.01 % sodium desoxycholate in 0.1 M phosphate buffer pH 7.3 and 20 mM Tris-HCl pH 7.3). For tissues showing high endogenous β-galactosidase activity (adrenal and mammary glands) 5-bromo-3-indolyl-β-D-galactopyranoside (bluo-gal; Sigma-Aldrich) was used as substrate in place of x-gal. Tissues were then washed in phosphate-buffered saline, dehydrated, and made transparent as previously described (Rentschler et al. 2001). To observe x-gal staining at a cellular level, tissue was fixed, stained with x-gal as above, dehydrated in a graded series of methanol, cleared with Histoclear (RA Lamb, UK) and paraffin-embedded. Sections were cut at 10 μm thickness, mounted on superfrost slides (VWR, UK), heated to 60°C for 1 h and stored at room temperature. Structures were identified from serial sections.

### Immunohistochemistry

Immunohistochemistry was used to visualize mouse Nat2 protein in 10 μm sections of e 11.5 embryos. Slides were dewaxed for 15 min in Histoclear and rehydrated through a graded alcohol series. Immunohistochemical staining was visualized using fast red-conjugated alkaline phosphatase (Dako En Vision, Dako, UK), following the manufacturer's instructions. Endogenous alkaline phosphatase activity was inhibited with a 15-min incubation in 20% acetic acid. Sections were probed for 4 h at 20°C with polyclonal anti-Nat2 antibody 184 as previously described (Stanley et al. 1998). Adjacent sections were incubated with preimmune serum (at 1:400) as controls. Monoclonal antibody 3A10, against neurofilament-associated antigen (Yamada et al. 1991) was used at a dilution of 1 in 10. After immunostaining, sections were counterstained with haematoxylin, air-dried and oil mounted.

## Results

### Mouse Nat2 expression in the developing neuroendocrine system

The activity of the *Nat2* promoter can be visualized in *Nat2* null mice in which the *lacZ* gene disrupts the *Nat2* coding region. This is achieved by monitoring the activity of β-galactosidase (the *lacZ* gene product) using a colorimetric assay.

**Figure 1.**
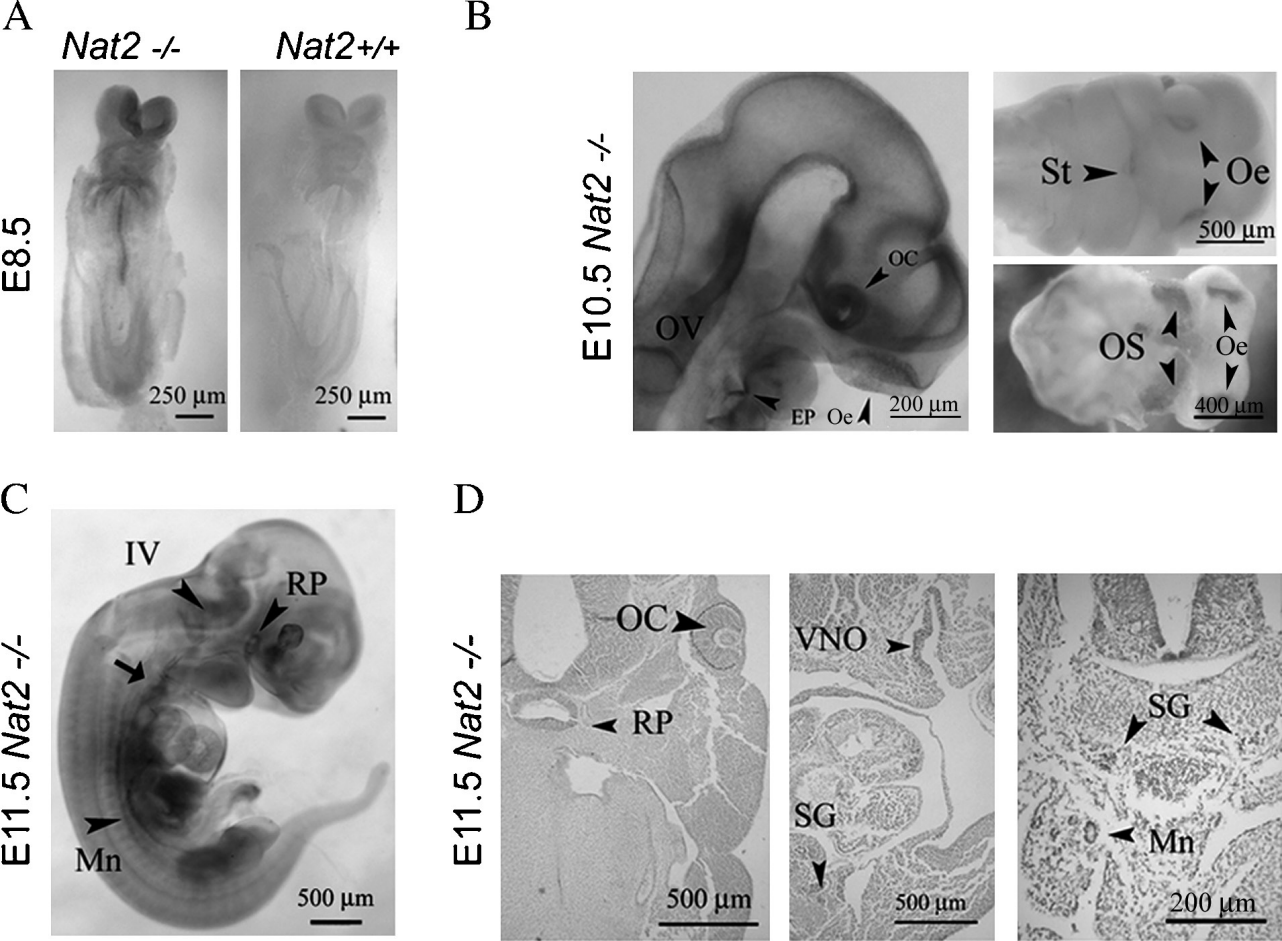
Mouse *Nat2* expression in the developing neuroendocrine system. (A) X-gal stained whole-mount e 8.5 embryos, with Nat2*null expression visualized by x-gal stain. Left panel: ventral view of *Nat2*^−/−^ embryo; right hand panel: *Nat2*^+/+^ sibling control for endogenous β-galactosidase activity. (B) Whole-mount preparation showing sensory placodes in *Nat2*^−/−^ embryos at e 10.5. Left panel: embryo made transparent with benzyl alcohol/benzoylbenzoate, arrowheads indicate optic cup, olfactory epithelium and epibranchial placode; right panels: ventral views of partially dissected whole-mount preparations showing primitive mouth (stomatodeum), olfactory epithelia and optic stalk (arrowheads). (C) Whole-mount preparation of e 11.5 embryo. Arrowheads indicate Rathke's pouch, floor of the IVth ventricle, and mesonephric ducts, arrow indicates developing nodose ganglion. (D) Sections of x-gal stained e 11.5 *Nat2*^−/−^ embryos, haematoxylin counterstained, showing components of the neuroendocrine system; Rathke's pouch, the retina, vomeronasal organ, sympathetic ganglia and mesonephric tubules (arrowheads). Scale bars: (A) 250 μm, (B–D) 500 μm. OV, otic vesicle; OC, optic cup; Oe, olfactory epithelium; EP, epibranchial placode; St, stomatodeum; OS, optic stalk; IV, fourth ventricle; RP, Rathke's pouch; VNO, vomeronasal organ; SG, sympathetic ganglion; Mn, mesonephric tubule. Colour available online.

*Nat2* gene expression was visualized via the blue product generated by β-galactosidase activity in *Nat2*^−/−^ embryos, over embryonic days e 8.5–11.5 (Figure 1). During the period of neural tube closure, at e 8.5, *Nat2* expression was detectable in the neural tube and in the neural crest cells migrating towards the developing heart (Figure 1A). Later, at e 10.5 *Nat2* expression was visible in the developing peripheral nervous system and parts of the central nervous system, more specifically, in the olfactory placodes, the otic vesicle, the epibranchial placodes and the floor of the fourth ventricle (Figure 1B). *Nat2* expression also extended laterally from the optic cup along the ventral surface of the optic stalk and anteroposteriorly along the floor plate. At e 11.5, in addition to continued expression in the developing peripheral and central nervous system, *Nat2* was expressed in the hypothalamic-pituitary-adrenal axis, from Rathke's pouch, along the sympathetic chain to the developing urogenital ridge (Figure 1C, D), from which the adrenocortical cells originate (Schulte et al. 2007).

### Nat2 protein localization in the developing neuroendocrine system and Nat2 enzyme activity during embryogenesis

To establish whether *Nat2* promoter use, as assayed by x-gal staining in *Nat2*^−/−^ embryos, is an indicator of productive gene expression, giving rise to functionally active protein in embryonic *Nat2*^+/+^ tissues, we performed immunohistochemical and biochemical analyses using Nat2-specific antibodies and Nat2-specific substrates.

**Figure 2.**
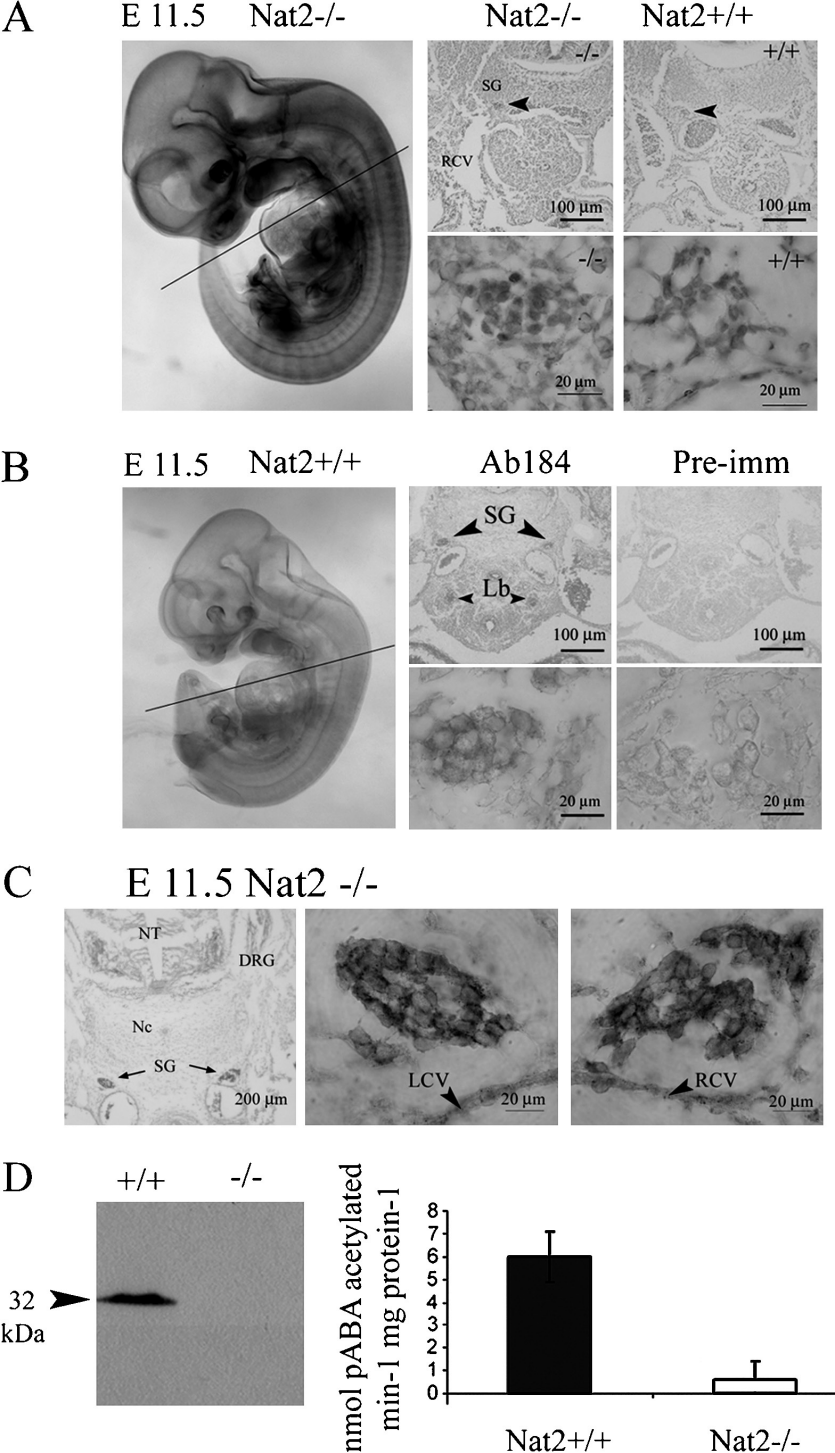
Nat2 protein distribution and enzymatic activity at embryonic day 11.5. (A) *Nat2*^−/−^ embryo at e 11.5, x-gal stained and prepared as whole mount. Panels show x-gal stain in transverse sections at the level of the heart (plane indicated on whole-mount preparation) in *Nat2*^−/−^ and *Nat2*^+/+^ siblings; blue x-gal staining in the sympathetic ganglia of *Nat2*^−/−^ embryos, viewed also under high magnification, indicates *Nat2* gene expression. (B) Whole-mount preparation of x-gal stained *Nat2*^+/+^ sibling illustrating the low level of endogenous β-galactosidase. Panels show transverse sections through a *Nat2*^+/+^ embryo at the level of the heart, as indicated on the whole-mount preparation. Sections were probed with mouse Nat2-specific polyclonal antibody 184 (Ab184) (Stanley et al. 1998) or preimmune serum (Pre-imm) as indicated and visualized using alkaline phosphatase (AP)-conjugated fast red. Red stain viewed also under high magnification indicates the presence of Nat2 protein. SG, sympathetic ganglia; RCV, right cardinal vein; Lb, lung bud. (C) Transverse section through a *Nat2*^−/−^ embryo fixed and stained with x-gal (blue) to reveal *Nat2* expression and subsequently treated with a monoclonal antibody to neurofilament, visualized with AP-conjugated fast red to distinguish neuronal (red) from glial cells. Centre and right panels are high magnification views of the sympathetic chain on the left and right side of the embryo indicated with black arrows (left panel). (D) Western blot of homogenates of e11.5 *Nat2*^+/+^ and *Nat2*^−/−^ embryos testing for the presence of Nat2 protein in *Nat2*^+/+^ and *Nat2*^−/−^ embryos (*n* =6 per genotype). *In vitro* acetylation assays using Nat2-specific substrate *para*-aminobenzoic acid (pABA) and acetyl coenzymeA, assaying Nat2-dependent acetylating activity in homogenates of e 11.5 *Nat2*^+/+^ and *Nat2*^−/−^ embryos (*n*>6 per genotype for each of two independent experiments). Tissues from *Nat2*^+/+^ and *Nat2*^−/−^ embryos show a significant difference in pABA-specific acetylation activity (*p*=0.03). NT, neural tube; Nc, notochord; DRG, dorsal root ganglion; SG, sympathetic ganglion; LCV, left cardinal vein; RCV, right cardinal vein. Colour available online.

*Nat2* promoter use is indicated by blue stain (Figure 2A), which shows robust expression in the developing sympathetic chain. The presence of immunoreactive mouse Nat2 in the developing sympathetic chain was probed in *Nat2*^+/+^ embryos by immunohistochemistry using a Nat2-specific polyclonal antibody (Stanley et al. 1996) (Figure 2B). To establish whether *Nat2* is expressed within neuronal or glial lineages, neurons were identified by immunohistochemistry, using a monoclonal antibody to neurofilament, visualized by fast red-conjugated alkaline phosphatase (Figure 2C). The lack of significant overlap of red (neurofilament) and blue (x-gal) staining, and partitioning of the x-gal stain in neurofilament-negative cells indicates that within the sympathetic chain, *Nat2* is expressed in glial cells (Figure 2C). Indications of robust *Nat2* gene expression in the developing sympathetic ganglia assayed using blue x-gal stain in sections of *Nat2*^−/−^ embryos were confirmed by the presence of Nat2 protein in sections of *Nat2*^+/+^ siblings. The presence of mouse Nat2 protein in whole embryos at e 11.5 was further demonstrated by Western blot analysis, and *N-*acetyltransferase catalytic activity assayed *in vitro* using the mouse Nat2-dependent substrate pABA (Figure 2D). Homogenates of *Nat2*^+/+^ embryos at e 11.5 *N*-acetylated pABA, demonstrating that Nat2 was enzymatically active at this stage. pABA acetylation was almost undetectable in *Nat2*^−/−^ embryos, confirming the functional deletion in the *Nat2* null mutant. Thus, in *Nat2*^+/+^ embryos, Nat2 protein was both present and enzymatically active in the developing neuroendocrine system from early in organogenesis.

### Nat2 expression and acetylation activity in adult tissues

#### Heart

**Figure 3.**
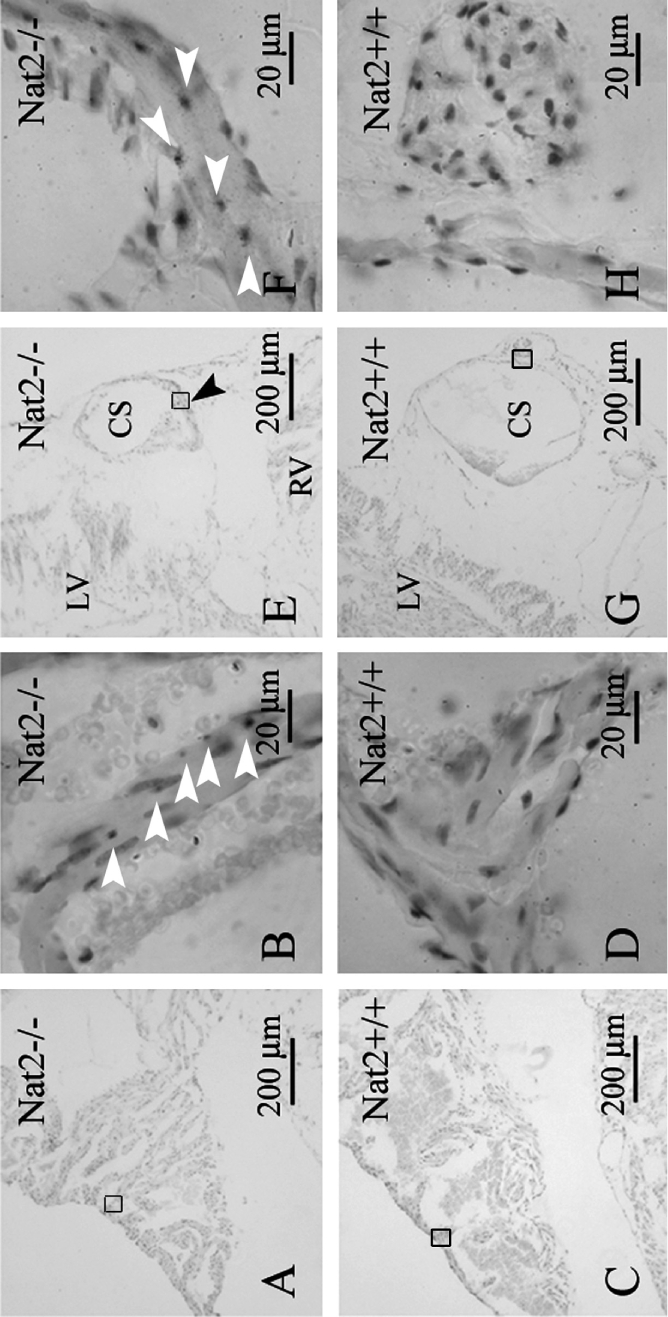
*Nat2* expression in adult heart. *Nat2* expression detected by x-gal staining in sections through the short axis of 8-week-old adult *Nat2*^−/−^ and control *Nat2* mouse hearts at the level of the tricuspid valve. (A–D) Right atrium; (E–H) coronary sinus (both left and right ventricular myocardium visible in (E). Site of *Nat2* expression are indicated by arrowheads in frame (F). All samples were counterstained with haematoxylin. Scale bars: (A, C, E, G) 200 μ m; (B, D, F, H) 20 μ m. LV, left ventricle; RV, right ventricle; CS, coronary sinus. Colour available online.

Nat2 expression in hearts taken from adult mice was also associated with autonomic innervation. *Nat2* expression was not detected in the ventricular myocardial cells, but was detected in glial cells interspersed with atrial myocardial cells (Figure 3A, B). *Nat2* expression was also evident in glial cells accompanying the sympathetic nerve along the coronary sinus (Figure 3E, F). No x-gal staining was observed in hearts isolated from *Nat2*^+/+^ mice (Figure 3C, D, G, H) indicating the lack of endogenous β-galactosidase activity and therefore the specificity of x-gal staining in adult *Nat2*^−/−^ tissue (Figure 3A, B, E, F). These observations are compatible with the continued expression of *Nat2* through the development of the sympathetic nervous system into adulthood.

#### Adrenal gland

**Figure 4.**
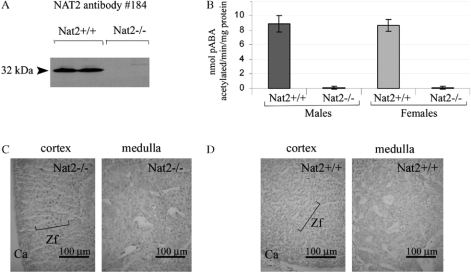
*Nat2* expression and catalytic activity in adrenal glands. (A) Western blot of adrenal gland homogenates from male and female adult *Nat2*^+/+^ and *Nat2*^−/−^ mice probed with polyclonal antibody 184 at 1:4000 to detect *Nat2*. (B) Nat2 *N*-acetylation activity assayed *in vitro* using mouse Nat2-specific substrate para-aminobenzoic acid (pABA). Cytoplasmic homogenates from adrenal glands of adult male and female *Nat2*^+/+^ and *Nat2*^−/−^ mice were assayed for their ability to N-acetylate pABA. pABA acetylation rates in adrenal glands from *Nat2*^+/+^ and *Nat2*^−/−^ are significantly different (*p* =2 × 10^−5^, *n* = 9 per sex and per genotype tested in three independent assays). Samples from males and from females do not show significant differences in acetylation rate. (C and D) Transverse sections through bluo-gal stained adrenal glands isolated from adult *Nat2*^−/−^ (C) and *Nat2*^+/+^ (D) female mice. Ca, capsule; Zf, zona fasciculate. Scale bars: 100 μm. Colour available online.

As components of adrenal gland function can be considered to form part of the neuroendocrine system, we have analyzed Nat2 protein levels and Nat2 catalytic acetylation activity in adrenal glands of adult mice (Figure 4). Adult mouse adrenal glands showed significant levels of pABA *N*-acetylating activity in both males and females (Figure 4B). In adult *Nat2*^−/−^ mice, functional deletion of the *Nat2* gene was confirmed by lack of immunoreactive Nat2 protein (Figure 4A) and pABA *N*-acetylating activity *in vitro* (Figure 4B). *Nat2* gene expression was detected, by histochemical staining via the *lacZ* marker gene in *Nat2*^−/−^ mice, both in the glucocorticoid-synthesizing zona fasciculata layer of the adrenal cortex as well as being scattered throughout the medulla (Figure 4C; and control for endogenous β-galactosidase activity, Figure 4D).

#### Oviduct

**Figure 5.**
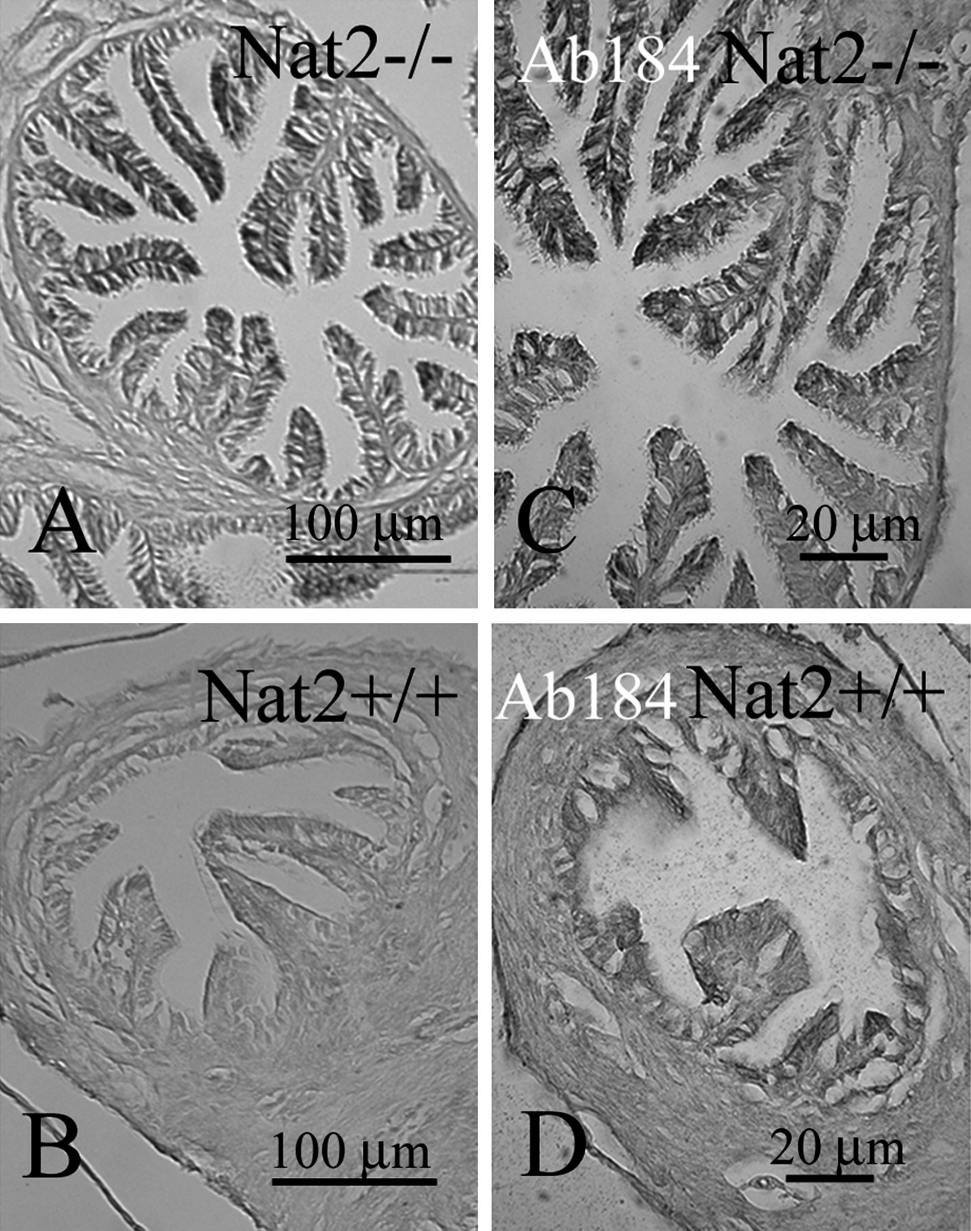
*Nat2* expression in oviduct epithelia. Transverse sections through bluo-gal stained oviducts of adult female *Nat2*^−/−^ and *Nat2*^+/+^ mice, analyzed for *Nat2* expression and Nat2 protein; blue stain indicates *Nat2* expression, detectable in *Nat2*^−/−^ tissues (A and C), red stain indicates Nat2 protein in*Nat2*^+/+^ tissues (D). (A and C) Sections through bluo-gal stained oviduct isolated from *Nat2*^−/−^ mice. (B and D) Sections through bluo-gal stained control *Nat2*^+/+^ mice. (C and D) After x-gal staining, sectioning and dewaxing, sections were probed with polyclonal anti-Nat2 antibody 184, visualized with alkaline phosphatase-conjugated fast red, to confirm the presence of Nat2 in *Nat2*^+/+^ tissues. Scale bars: (A, B) 20 μm; (C, D) 100 μm. Colour available online.

We investigated the expression of *Nat2* in normal mouse oviduct (Figure 5), a tissue responsive to neuroendocrine signalling. Use of the *lacZ* gene in *Nat2*^−/−^ mice to monitor *Nat2* expression, showed high levels of *Nat2* expression in the luminal epithelium (Figure 5A, C). This pattern of staining was not detected in luminal cells from *Nat2*^+/+^ mice, which lack the bacterial β-galactosidase. The samples in Figure 5 were initially stained with bluo-gal, to monitor *Nat2* expression via the *lacZ* gene in tissues from *Nat2*^−/−^ mice. Samples shown in Figure 5C and D were subsequently probed with a polyclonal antibody raised against mouse Nat2 protein. Fast red stain in these panels shows that Nat2 protein was present in the luminal epithelium of oviducts from *Nat2*^+/+^ mice, but absent from the luminal epithelium of oviducts from *Nat2*^−/−^ mice, which lack functional Nat2 protein.

## Discussion

Using *Nat2* knockout transgenic mice to model human *NAT1*, we have extended previous studies mapping the mouse *Nat2* expression pattern and acetylation activity in developing embryos. We found that *Nat2* was expressed in the sensory epithelia and epibranchial placodes, which give rise to the sensory neurons innervating the heart and digestive system (Kirby 1988), in the sympathetic chain and developing urogenital system. *Nat2* expression has previously been described in the developing heart (Stanley et al. 1998, Wakefield et al. 2005). In neonates, cells expressing high levels of *Nat2* occur in regions of the heart innervated by the peripheral nervous system (Wakefield et al. 2005). The location of *Nat2* expression in both the developing and adult heart, and punctate staining pattern (Figure 3) corresponds to that of *Sox 10*-expressing glial cells accompanying the autonomic innervation of the heart (Montero et al. 2002). In embryos, identification of cells differentiating along a neuronal pathway, using an anti-neurofilament antibody, positively identifies the sympathetic chain and suggests that *Nat2* is expressed in glial cells, as previously described (Stanley et al. 1998), further supporting a neuroendocrine expression pattern. *Nat2* expression in the developing sympathetic nervous system, which is largely derived from folate-sensitive neural crest cells is compatible with the proposed role of human NAT1 in folate metabolism (Minchin 1995). As in humans, we found that mouse adrenal glands showed substantial pABA *N*-acetylating activity. Within the adrenal glands *Nat2* expression was observed both in the neural crest-derived medulla and in the cortex. Since the adrenal cortex plays a major role in steroid hormone and glucocorticoid synthesis from cholesterol, *Nat2* expression in this region is consistent with a functional role in the adult and developing neuroendocrine system.

The presence of human *NAT1* in luminal cells within the gut, lung (Barker et al. 2006), bladder and kidney epithelia (Stanley et al. 1997), clearly indicates that its expression is widespread. In these epithelia, human *NAT1* may have an endogenous function, in addition to that of xenobiotic metabolism. We observed robust mouse *Nat2* expression and Nat2 protein in mouse oviduct epithelia, known to respond to endogenous and xenobiotic estrogens and *Nat2* expression and Nat2 activity has been previously demonstrated in rodent prostate tissue, which is also oestrogen responsive.

Previous studies in mice indicate that although the deletion of the *Nat2* gene is compatible with normal development, and most offspring are aphenotypic, the *Nat2* null allele gives rise to skewed sex ratios both on mixed (Cornish et al. 2003) and pure genetic backgrounds (Wakefield et al. 2006). These results indicate a gender-dependent phenotype, discernable at the population level. Such a phenotype could arise if *Nat2* gene mutations have opposing effects on male and female offspring, influencing sex ratios *in utero* and/or embryonic survival. Sex steroids (both oestrogen and testosterone) and corticosteroids are synthesized and have biological roles in both sexes and changes in the balance of these steroids may influence offspring sex ratios. The pattern of robust *Nat2* expression and gender-dependent effect of the *Nat2* null mutation implicate *Nat2* function in the sexual dimorphism of neuroendocrine function.

**Figure 6.**
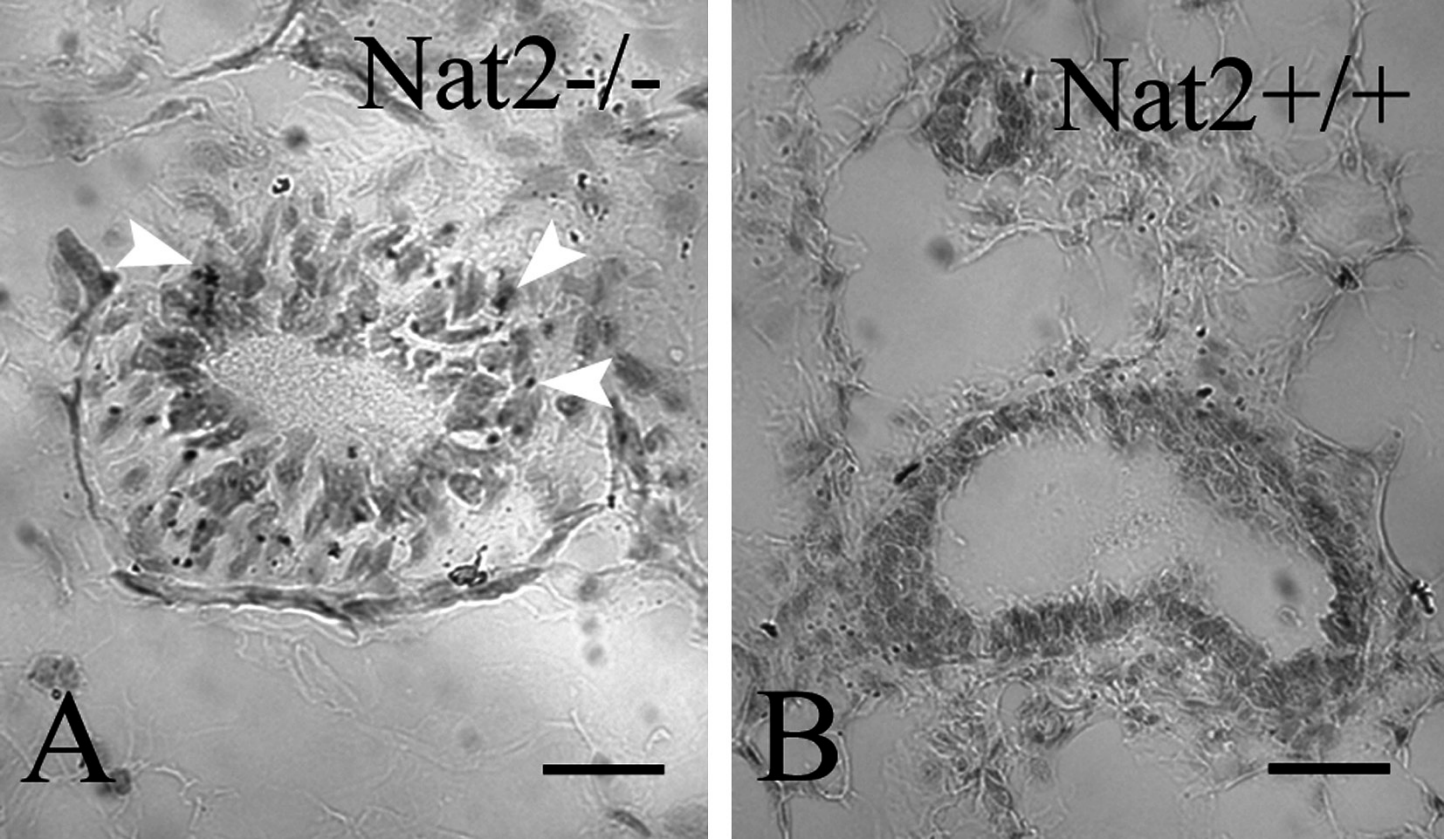
*Nat2* expression in mouse mammary gland epithelia. (A and B) Sections through bluo-gal stained mammary gland isolated from *Nat2*^−/−^ (A) and *Nat2*^+/+^ (control) (B) adult virgin female mice. Blue stain (bluo-gal β-galactosidase substrate) indicates *Nat2* expression, detectable in *Nat2*^−/−^ tissues, highlighted with arrow heads. Scale bars: (A, B) 20 μm. Colour available online.

A correlation between human *NAT1* overexpression and oestrogen receptor status in breast cancer tissues has been described (Adam et al. 2003) which implicates human NAT1 in oestrogen-positive breast tumours and in relapse-free survival prognosis (Bieche et al. 2004). Using *Nat2*^−/−^ mice, we have been able to demonstrate low levels of expression in mouse mammary gland (Figure 6), indicating that in mice as in humans low levels of expression of mouse *Nat2*/human *NAT1* genes is associated with normal growth of mammary epithelia. Our findings, using mouse Nat2 to model human NAT1 function, indicate a novel aspect to the pattern of expression of mouse *Nat2*/human *NAT1* during embryogenesis and will be important in understanding the role of the human *NAT1* as a potential biomarker in breast cancer.
